# Organic Spin‐State Photoswitches

**DOI:** 10.1002/anie.202512691

**Published:** 2025-08-22

**Authors:** Takuma Miyamura, Joël Schlecht, Oliver Dumele

**Affiliations:** ^1^ Department of Organic Chemistry Albert‐Ludwigs‐Universität Freiburg Alberstrasse 21 79104 Freiburg Germany; ^2^ Department of Chemistry and Biochemistry University of Cologne Greinstrasse 4 50939 Cologne Germany

**Keywords:** Chromophores, Diradicals, Molecular magnetism, Photoswitches, Spin materials

## Abstract

Organic spin‐state photoswitches represent an emerging class of molecular systems capable of reversibly modulating electronic spin states using light. This review provides a comprehensive overview of the fundamental mechanisms underlying two principal switching modes: photoconformational and photochemical. Emphasis is placed on their structural design, magnetic behavior, and the methods used for their characterization, including electron paramagnetic resonance (EPR) and UV–vis spectroscopy. Selected examples illustrate the ability of these systems to control spin‐polarized states, alter magnetic exchange interactions, or switch between singlet and triplet spin states upon photoirradiation. The review also discusses challenges related to thermal stability, bistability, and operation under ambient conditions. Emerging strategies for integrating photoswitches with persistent radicals or redox‐active units are highlighted for their potential in molecular electronics and quantum information science. Notably, light‐induced diradical species in such systems are gaining recognition as molecular qubits, offering long coherence times and spectroscopic addressability essential for quantum gate operations. By exploring design principles and technological implications, this review underscores the unique position of all‐organic spin‐state photoswitches at the intersection of photochemistry, magnetism, and quantum materials development.

## Graphical abstract

Spin‐state photoswitching has become a field of broad interest in the last decades due to unique physical and chemical properties accessible from controlling the spin state of a molecular entity. This review gives an overview of the different types of materials and classifies them into photoconformational and photochemical switches depending on the mechanism of action. It highlights milestones in the research over the years and gives a technological perspective for the future of spin‐state photoswitches.

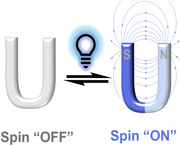

## 1 Introduction

Precisely controlling the chemical and physical properties of molecular entities is undoubtedly one of the longest‐standing challenges and motivations for scientists. Tailored action–response systems can be designed for the various demands faced. A particularly interesting molecular property is the electronic spin state *S*, which dictates the spin multiplicity 2*S* + 1 of an atom, molecule, or complex.^[^ ^1^, ^2^ ^]^ Most organic molecules and main‐group inorganic compounds have a closed‐shell electronic configuration (*S* = 0). Transition metal complexes often exhibit differing spin multiplicities according to the ligand field theory, with low‐spin or high‐spin configurations depending on the coordination sphere.^[^ ^3^ ^]^ Reversibly changing the electronic configuration can be achieved by spin‐crossover complexes. While this is a timely field with many contributions, systems with the spin located on metals have been extensively reviewed.^[^ ^4^, ^5^, ^6^, ^7^, ^8^, ^9^, ^10^ ^]^

**Figure 1:**
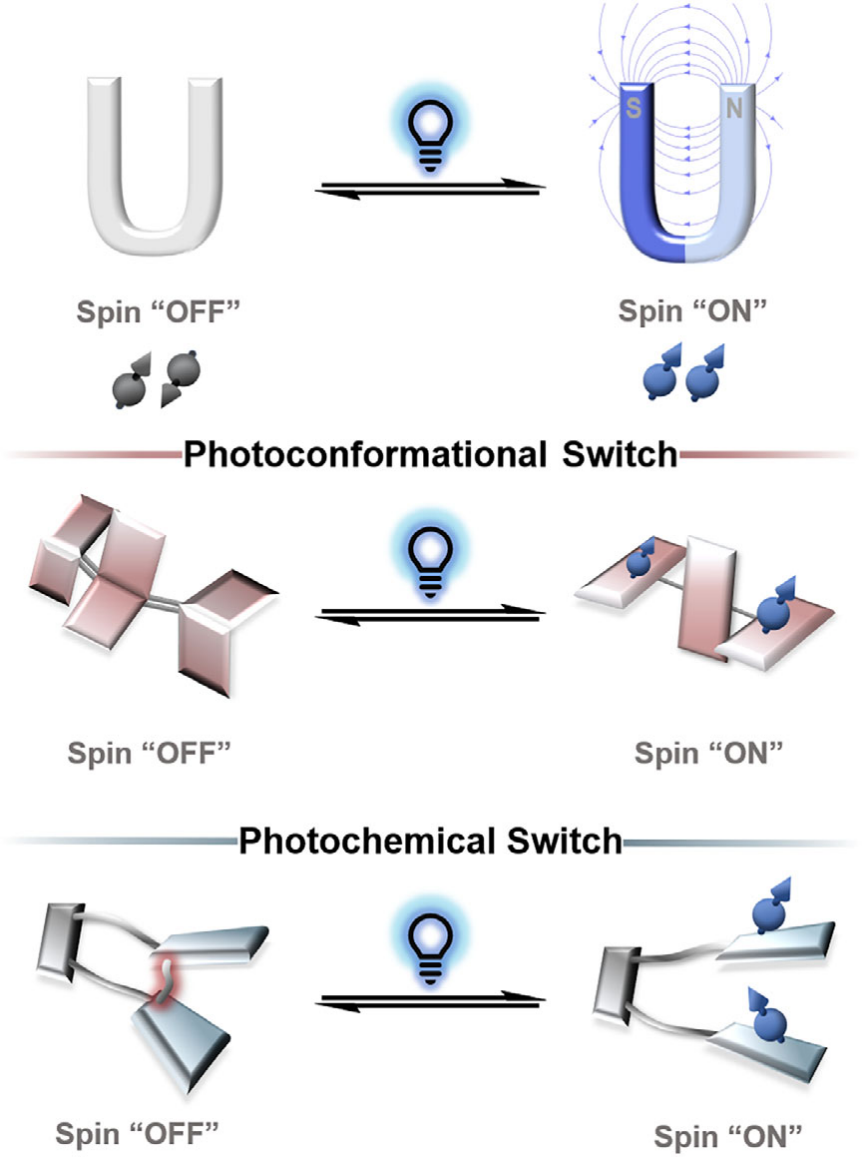
General principles of spin‐state photoswitching.

Stable and purely organic molecules are usually in a locked closed‐shell electronic configuration. π‐Systems exhibiting open‐shell character have been developed, and their electronic structure is often driven by the principles of aromaticity.^[^ ^11^, ^12^, ^13^, ^14^, ^15^, ^16^, ^17^, ^18^ ^]^ An exemption to the general closed‐shell trend in organic molecules is non‐Kekulé hydrocarbons, some of which exhibit stable open‐shell diradical structures under ambient conditions.^[^ ^19^, ^20^, ^21^, ^22^, ^23^ ^]^ In addition, a number of stable organic aminoxyl radicals,^[^ ^24^, ^25^, ^26^, ^27^ ^]^ triphenylmethyl radicals,^[^ ^28^, ^29^, ^30^ ^]^ allylic radicals,^[^ ^31^, ^32^, ^33^, ^34^, ^35^ ^]^ and other permanently open‐shell structures are known.^[^ ^36^, ^37^, ^38^, ^39^, ^40^, ^41^ ^]^ Changing the spin state of organic systems is challenging, especially when attempted in a reversible fashion triggered by an external stimulus (Figure 1). The versatility of an all‐organic design, however, allows for addressing tailored properties, such as spin–spin distance and specific tuning of the trigger (such as wavelengths) for the spin switch, giving lightweight, nontoxic, and more flexible materials that exceed the structure space of metal‐containing systems.

This review aims to give a comprehensive overview of all‐organic spin‐state photoswitches, with their experimental and computational evaluation, over the last decades. We define organic spin‐state photoswitches as a class of photochromic molecules bearing at least two different interconvertible (meta)stable spin states. The focus of this review is on spin‐state changes induced by local transformation and internal motion of the molecular scaffold, but not by phase transitions.^[^ ^42^, ^43^, ^44^ ^]^ Additionally, we only include examples of reversible switches for which at least one directional trigger is light and refer to a selection of other reviews covering different types of organic open‐shell materials.^[^ ^45^, ^46^, ^47^, ^48^, ^49^, ^50^ ^]^ Neither photophysical phenomena that reversibly change their spin state in the transient excited states^[^ ^51^, ^52^, ^53^ ^]^ nor systems that generate charge‐separated states upon photoinduced proton transfer^[^ ^54^ ^]^ or upon electron transfer^[^ ^55^, ^56^, ^57^ ^]^ are included. Moreover, to uphold the depth of this review, we limit the discussion to the range of the molecular level. As this scientific area is relatively young, a perspective on possible future materials and their implementations for novel applications is outlined. The breadth of future potential technologies would require a wide range of properties. For instance, applications in quantum information science (quantum sensing/computing) are compatible with a low‐temperature range of switching and therefore require only a small thermal barrier between the (meta)stable states. On the other hand, if everyday use in common devices or in biomedical applications is targeted, visible light stimuli and high thermal barriers with long half‐life times of the metastable state are desirable. In this review, we address the molecular design principles based on these criteria.

The switches are categorized into two groups with several representative examples: I) Spin‐state switching via a photo‐conformational change, along with a configurational isomerization of the molecule involving twisting or *cis*–*trans* isomerization of double bonds (Figure 1). II) A photochemical reaction (homolytic bond cleavage or pericyclic reaction) that results in new connectivity of the organic scaffold causing the spin‐state change (Figure 1). Both categories have prominent examples and their design, and switching is discussed throughout this review.

## 2 Methods for the Analysis of Spin‐State Switches

To date, a number of methods for the characterization and analysis of spin‐state switches have been established. The definite proof of the change of the electronic configuration upon photoirradiation relies on a selection of experimental and theoretical methods. As the interest in the field and understanding of the underlying principles increase, we expect to see a broadening of the methods used in the future to characterize these materials in even more detail.

### 2.1 Experimental Methods

**Figure 2:**
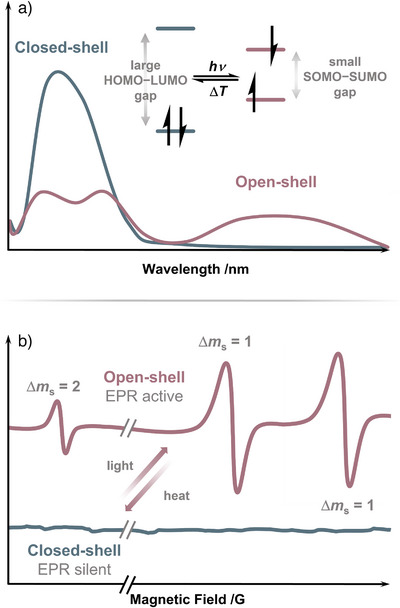
a) Sketch of an idealized UV–vis spectrum before and after irradiation with relevant orbital levels; b) Sketch of an idealized EPR spectrum before irradiation (silent) and after irradiation (triplet).

Undoubtedly the most common and straightforward experimental indication of spin‐state switching is irradiation monitored by UV–vis spectroscopy in solution or solids.^[^ ^58^, ^59^, ^60^, ^61^ ^]^ The generation of an open‐shell species upon irradiation is often accompanied by the formation of broad long‐wavelength absorption bands due to the narrow SOMO–SUMO energy gap of the single occupied molecular orbitals (Figure 2a). Ceasing irradiation or increasing the temperature or irradiation with a different wavelength then leads to the reverse, and the initial spectrum is regained.

**Figure 3:**
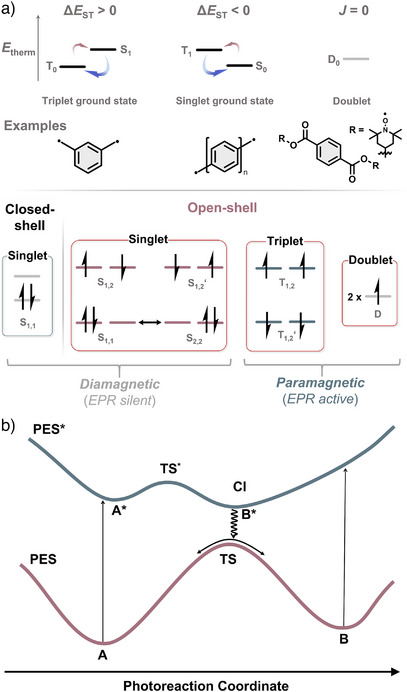
a) Thermal energy distribution of open‐shell ground and excited states with molecular examples. Electronic configurations of closed‐shell and open‐shell spin systems; b) Idealized potential energy surfaces for a typical photoreaction.

Electron paramagnetic resonance (EPR) spectroscopy is a valuable tool in the characterization and analysis of spin‐state switches. For example, a molecular system exhibits a closed‐shell singlet electronic configuration and therefore no unpaired electrons, hence showing no EPR signal. Irradiation of that system leads to a phototransformation generating one unpaired electron or two isolated monoradicals (biradical). The EPR spectrum shows a doublet signal. Examples for such simple changes are discussed below.^[^ ^58^, ^59^, ^62^ ^]^ In many cases, however, the observed EPR signals are much more complex, as they are usually observed for two non‐isolated radicals (diradical(oid)s), resulting in a triplet pattern. An idealized spectrum of a triplet diradical in the solid state shows three peaks, a low‐intensity peak at half field corresponding to the formally forbidden Δ*m*_s_ = 2, and two intense peaks arising from the zero‐field splitting and corresponding to Δ*m*_s_ = 1 (Figure 2b).^[^ ^63^, ^64^, ^65^ ^]^ In some cases, the observed spectra cannot be clearly assigned to one spin state, but rather the change of interaction between two unpaired electrons in a single molecule is of interest.^[^ ^66^, ^67^, ^68^, ^69^, ^70^ ^]^ The change of interaction between two radicals is described with the electron exchange integral *J*. If *J* = 0, the two radicals are isolated and can be understood as two monoradicals, resulting in a doublet spin state. The molecule is called biradical.^[^ ^1^, ^71^ ^]^ If *J* ≠ 0, the radicals interact with each other, generally giving a triplet ground state with a thermally excited singlet state according to Hund’*s* rule (Figure 3a).^[^ ^72^ ^]^ The molecule is called diradical.^[^ ^71^, ^73^ ^]^ If *J* is close to zero but not negligibly small, the ground state is usually a singlet with a thermally accessible triplet excited state (Figure 3a). The molecule is called diradicaloid (or biradicaloid).^[^ ^71^, ^73^ ^]^ Varying temperature EPR (VT‐EPR) is used to determine the spin multiplicity in the ground state and therefore differentiate between diradicals and diradicaloids.

Besides UV–vis and EPR spectroscopy, other experimental techniques have been used to analyze and characterize spin‐state switching materials. ^1^H NMR spectroscopy,^[^ ^62^ ^]^ single‐crystal X‐ray diffraction (SCXRD),^[^ ^65^, ^74^ ^]^ and superconducting quantum interference device (SQUID)^[^ ^75^ ^]^ measurements have been used in rare cases to give further insights. These methods are, however, not routinely applied. For example, in ^1^H NMR spectroscopy as a common method for organic chemists, the absence of proton resonances in proximity to the radicals indicates a paramagnetic species. X‐ray crystallography gives unique insight into the solid‐state geometry of radicals or open‐shell species. Other magnetization methods (such as SQUID) can reveal crucial information about a material's magnetic properties, including Curie temperature (*T*_c_), spin dynamics, magnetic anisotropy, and the presence of magnetic interactions.

### 2.2 Theoretical Methods

Each electronic state of an organic molecule can be represented by a potential energy surface (PES, Figure 3).^[^ ^76^ ^]^ Upon absorption of light, the molecule transitions from the ground state (A) to the electronically excited state (A*). A fraction of these excited species undergoes structural reorganization, relaxing to a saddle point within the excited‐state PES*. Via a conical intersection (CI), the molecule relaxes down to the ground state and distributes between A and B, populating B over time. Photoswitches are characterized by a (thermal) activation barrier (TS) that separates the isomeric forms, A and B. The topography of the excited‐ and ground‐state PES determines the switching behavior, with some systems exhibiting purely photochemical reversibility, others relying on thermal back reaction, and certain cases displaying both mechanisms‐ in tandem.^[^ ^77^, ^78^ ^]^

What distinguishes spin‐state switching molecules from persistent closed‐shell organic systems is the existence of an open‐shell (meta)stable state. To rationally design systems and to understand experimental results, investigations on the electronic configuration of the metastable states are essential. In general, quantum computation in the ground state of closed‐ and open‐shell forms can reproduce experimental results in good quality with low computational costs. Properties that cannot be accessed through experiments can be calculated. For example, structural features of metastable states that cannot be isolated. In this review, instead of going deep into a discussion on the theoretical background of quantum calculations, we aim to provide a guideline on how to simulate systems for organic chemists, with some examples in the Supporting Information.

The most intriguing question for the molecular system is the nature of the spin state in each form. Electronic configuration of closed‐shell states and open‐shell triplet states (T_1,2_, T_1,2′_) are generally calculated with single reference (un)restricted density functional theory (U)DFT—a calculation that can be run by most organic chemists nowadays. However, describing the open‐shell singlet states, both in the ground and excited states, is more challenging: Three electronic configurations are possible (S_1,2_, S_1,1_, and S_2,2_, Figure 3a), where the above‐mentioned single reference methods do not provide reliable results.^[^ ^71^, ^79^ ^]^ Consequently, calculation of Δ*E*_ST_ using such methods has been problematic. To address this issue, multireference models for quantum computations, such as complete‐active‐space self‐consistent‐field (CASSCF) methods or multireference coupled cluster methods, are often utilized to describe theoretically accurate properties of singlet diradical(oid)s.^[^ ^80^, ^81^ ^]^ Nevertheless, these multireference computations demand large computational costs.

Alternatively, approaching open‐shell singlet states using DFT methods has attracted many scientists’ attention.^[^ ^82^, ^83^ ^]^ Despite DFT being a single‐reference computational method, its accuracy and convenience in return for small computational resources should not be neglected. In the 1980s, the teams of Noodleman and Yamaguchi demonstrated broken‐symmetry (BS) Kohn–Sham methods to reproduce geometry and electronic properties of singlet diradicaloid systems by mixing triplet and singlet states.^[^ ^84^, ^85^, ^86^, ^87^ ^]^ Their development enabled rapid and efficient estimation of the energy and electronic configuration of singlet‐diradical(oid) systems.

For computational cost efficiency, it is common to first optimize the geometries A, B, and TS of spin‐state photoswitches in all expected spin states utilizing the (BS)‐DFT level of theory (Figure 3). The relative energies of TS to A and B allow to estimate the thermal stability of the system. Further on, single‐point computations are followed to predict electronic properties, such as optical absorption bands (time‐dependent DFT), aromaticity evaluations,^[^ ^88^, ^89^, ^90^, ^91^, ^92^, ^93^, ^94^, ^95^, ^96^ ^]^ EPR spectra,^[^ ^97^ ^]^ or using multireference computations for diradical indices and singlet–triplet energy gaps (Δ*E*_ST_).^[^ ^98^ ^]^ Additionally, spin density maps and molecular orbitals are often reported to rationalize the experimental measurements and their photochemical mechanisms.

## 3 Photoconformational Spin‐State Switches

**Figure 4:**
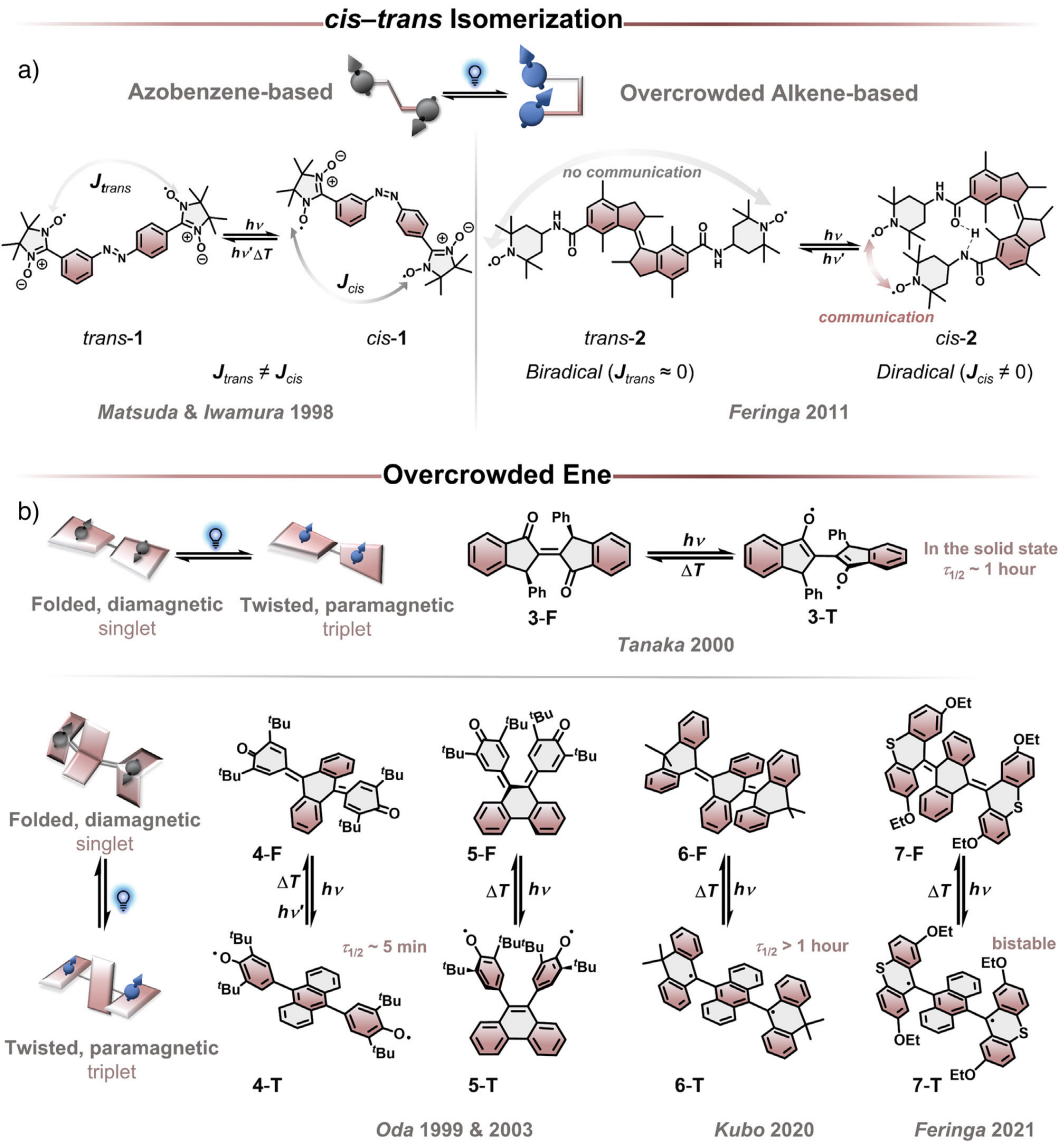
Photoconformational switches based on a) *cis*–*trans* isomerization and b) overcrowded ene.

Organic spin‐state photoswitches reported thus far can be categorized into two distinct groups, as described in the introduction. The first category comprises photoconformational switches, in which a change in spin state is triggered by a photoinitiated conformational and configurational isomerization while maintaining the overall connectivity. Such transformations are realized by *cis–trans* photoisomerization (Figure 4a) or strain‐induced bond twisting by isomerization of overcrowded double bonds (Figure 4b). While the configurational isomerization causes the spinstate switching based on the electronic structure, the conformational change is its unique characteristic.

Photoinduced isomerizations are a powerful tool for controlling molecular properties,^[^ ^82^ ^]^ particularly in spin‐state switching systems. One such approach relies on *cis–trans* photoisomerization, where a photochromic unit undergoes a geometric rearrangement upon light irradiation. This alters the distance between the persistent radical units, resulting in different magnetic interactions (Figure 4a). When the distance between two radical units is large (>ca. 10 Å, depending on the efficiency of conjugation), the exchange interaction *J* comes close to zero and vice versa. This method enables the modulation of spin states and magnetic coupling; however, both states can be paramagnetic to a different extent.

Matsuda, Iwamura and co‐workers reported a photoconformational spin‐state switch bearing two persistent nitronyl nitroxide radicals.^[^ ^75^ ^]^ They used a well‐established photochromic azobenzene as a ferromagnetic coupling unit of the geometrical *cis–trans* isomerization. Photoirradiation with 360–400 nm light of triplet *trans‐* **1** led to a photoreaction, which was confirmed by UV–vis spectroscopy. SQUID measurement followed by Bleaney–Bowers analysis revealed *trans*‐**1** possesses a triplet ground state, with 2 *J*/*k*_B_ = 8.36 ± 0.26 K (Δ*E*_ST_ = 16.6 ± 0.52·10^−3^ kcal mol^−1^). They confirmed a spectral change in the EPR after irradiation of *trans*‐**1** and cooling the temperature to 10 K, which indicates a change in the distance between the two unpaired electrons. The fine structure observed in *trans*‐**1** was replaced with a broad signal after irradiation. However, because the conversion of *trans*‐**1** to *cis*‐**1** was not quantitative, it was not possible to exclude the signals from the *trans* isomer. Therefore, the spin‐state switching of **1** was not conclusive.

Similar to this concept, Feringa and co‐workers reported a photoconformational spin‐state switch based on an overcrowded alkene (OA) with two tetramethylpiperidin‐1‐oxyl (TEMPO) radicals attached.^[^ ^99^ ^]^ In this system, TEMPO and the OA are linked in a non‐π‐conjugated manner to avoid losing photochemical switching efficiency. Light‐induced (irradiation with 312 nm light) *cis–trans* isomerization of the OA switched the through‐space magnetic interactions between the two unpaired electrons, leading to different EPR spectra. The EPR spectrum of *trans*‐**2** suggested nearly no spin interaction (*J* = 0) between the unpaired electrons, as a typical three‐line signal of TEMPO with a hyperfine coupling constant with ^14^N was observed. Meanwhile, the EPR spectrum showed substantial change upon photoirradiation with a larger coupling constant, resulting in a five‐line EPR signal. This strongly indicated the switching behavior from biradical (*trans*‐**2**) to diradical (*cis*‐**2**), both being paramagnetic. Designing spin‐state switches based on persistent radicals limits the switching between magnetic states, not allowing access to diamagnetic forms.

Incorporating molecular strain as a driving force for spin‐state switching has emerged as a compelling strategy in switching materials (Figure 4b). Unlike *cis–trans* isomerization, strain‐induced bond twisting relies on the release of strain and can be aided by the gain of aromaticity. Upon external stimulation—such as light, heat, or redox chemistry—these strained molecules undergo conformational changes that can stabilize open‐shell diradical states. A key feature of this molecular class is the presence of a closed‐shell state, enabling reversible photoswitching between diamagnetic and paramagnetic forms. This characteristic distinguishes them from photoswitches based on persistent radicals, where spin‐state interconversion does not involve a diamagnetic species. As a result, strain‐induced spin‐switching systems offer a unique avenue for designing tunable electronic and magnetic properties.

In the early 2000s, Toda and Tanaka reported an organic molecular system showing photochromic and photomagnetic properties in the solid state.^[^ ^59^, ^60^, ^61^ ^]^ Upon exposing the yellow crystals of folded enedione **3**‐F to sunlight, reddish‐purple crystals were obtained, which exhibited a triplet EPR signal in the solid state. The twisted dioxyl diradical **3**‐T had a half‐life of about 1 h in the crystalline form. However, no spin‐state switching was observed in solution. In the solid state, rotation around the overcrowded alkene is restricted, which leads to population of the diradical state **3**‐T. In contrast, in dichloromethane solution, enedione **3**‐F photoisomerized to the *cis* isomer with the aryl groups in the anti‐configuration while maintaining a diamagnetic form.

Similar to overcrowded alkenes, overcrowded aromatic enes (OAEs) are known to exhibit a conformational (and configurational) change upon a variety of external stimuli (heat, light, and electrochemical).^[^ ^50^, ^100^, ^101^, ^102^, ^103^, ^104^ ^]^ The two distinct conformations are separated by activation barrier(s), and the major driving force to form the diradical(oid) is the release of strain to the central polyaromatic unit, sometimes aided by the gain of aromaticity. While OAEs typically form a diradical through a thermal stimulus, four examples are presented in the next paragraphs that form a diradical upon light irradiation.

Early examples were reported by Oda and co‐workers in 1999, using a ter‐phenoquinone skeleton where two *p*‐phenoquinones are connected with an 1,6‐anthraquinoidal moiety that induced strain in the folded closed‐shell form **4**‐F.^[^ ^105^ ^]^ A triplet EPR signal was detected after irradiation of **4**‐F with a Xenon lamp in a degassed benzene solution, indicating the generation of a twisted open‐shell form of **4**‐T. The half‐life time of the EPR signal in the dark was 4 ± 1 min at 25 °C, correlating to an activation energy of Δ*G* ^‡^ = 21 kcal mol^−1^. The authors claim that besides the thermal back reaction from the diradical **4**‐T to **4**‐F, a photo‐triggered conversion is also feasible, which would make this the only example of a bidirectional photoswitch (p‐type photoswitch) reported in the literature. Interestingly, no switching behavior was observed when the central 1,6‐anthraquinoidal linking unit of **4**‐F was replaced with a less bulky *p*‐phenoquinoidal unit, showcasing the necessity of an activation barrier induced by over‐crowdedness.^[^ ^104^, ^106^ ^]^ Later in 2003, phenanthraquinoidal 5‐**F** was synthesized as a geometrical isomer of 4 and revealed a similar behavior; however, 5‐**T** did not switch back to **5**‐F with the trigger of light.^[^ ^107^ ^]^

It took almost two decades to significantly increase the half‐life time of the diradical state. Due to the pioneering contributions of Kubo, Nishiuchi, and co‐workers to the field of OAE,^[^ ^18^, ^108^ ^]^ a dimethyl‐anthryl (DMA)‐substituted anthraquinoidal scaffold **6**‐F with efficient spin‐switching behavior was developed.^[^ ^109^ ^]^ The folded diamagnetic form **6**‐F and the twisted diradical form **6**‐T were successfully isolated and further characterized by EPR and UV–vis spectroscopy. Upon photoirradiation, 6‐F undergoes a conformational change, releasing strain from the overcrowded fjord region, to adopt an orthogonal conformation in **6**‐T with two unpaired electrons, but the ground state spin multiplicity of **6**‐T remains elusive. Kinetic parameters were determined by monitoring UV–vis absorption of **6**‐T with a half‐life time of several hours and Δ*G* ^‡^ = 23.0 kcal mol^−1^ in solution at 300 K.

**Figure 5:**
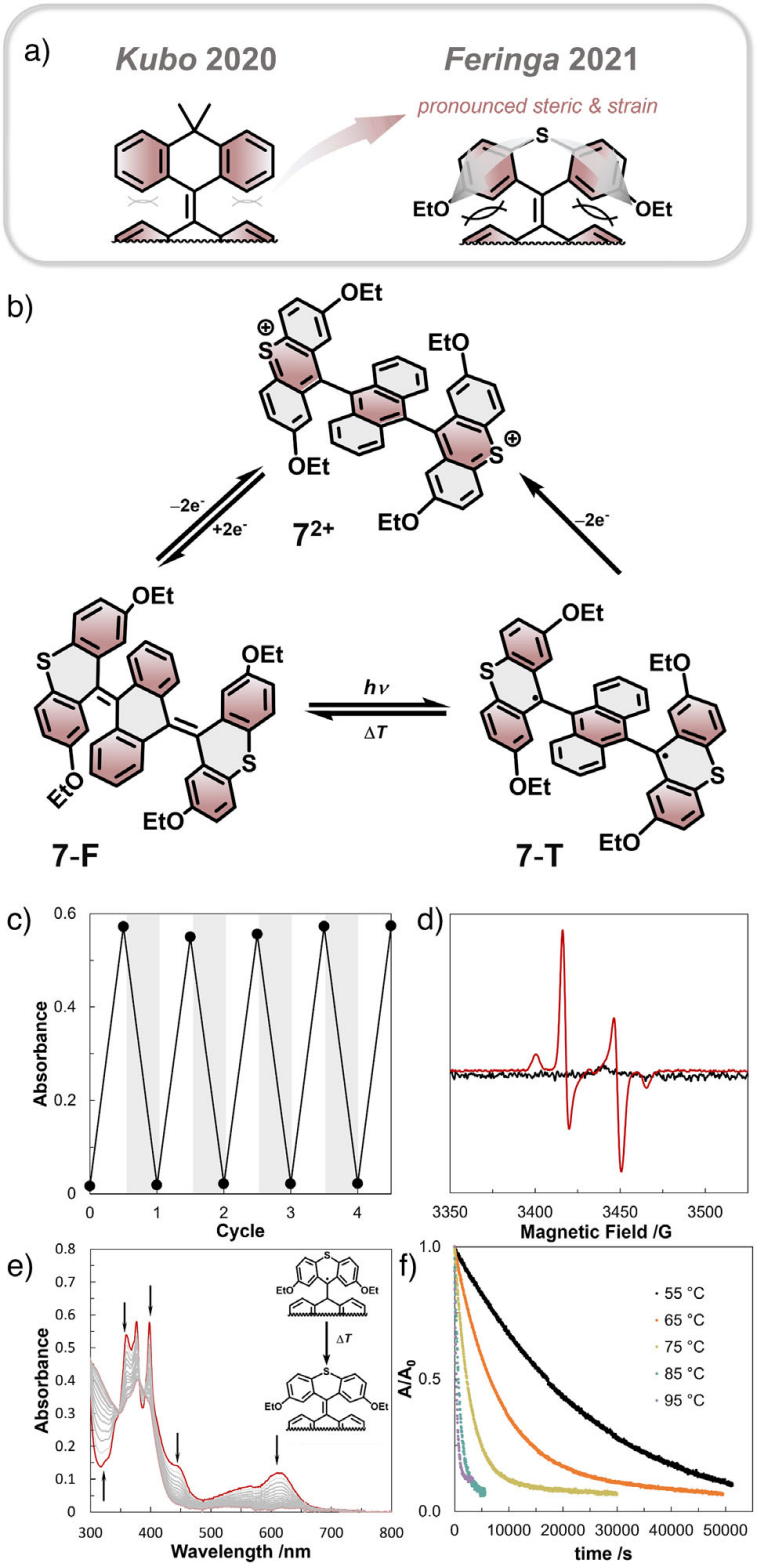
a) Substitution effect of dimethylanthryl and thioxanthyryl moieties; b) Photo/electrochemical spin‐state switching of **7**; c) Switching cycles of irradiating with 385 nm for 5 min (white areas) and heating at 90 °C for 1 h (grey areas) while monitoring absorbance at 615 nm; d) EPR spectra of 7‐F in frozen toluene at 77 K: before irradiation (black line) and after irradiation (red line); e) Series of UV–vis absorption spectroscopy in toluene showing the thermal decay of 7‐T at 55 °C: spectrum plotted every 1 h; f) Time profile of *A*/*A*_0_ at 615 nm at different temperatures. Figures edited and reprinted with permission of B. L. Feringa.^[^ ^110^ ^]^

Inspired by the previous work, bistable spin‐state switching was achieved by Feringa and co‐workers introducing additional steric bulk to increase the strain (Figure 5).^[^ ^110^ ^]^ Editing Kubo's scaffold with an additional sulfur atom as thiaxanthylidene led to a more rigid and sterically wider ene‐substituent. Additional substitution with ethoxy groups in the fjord region of the ene‐substituent resulted in an ideal steric bulk for room‐temperature bistability. Upon photoirradiation of the folded closed‐shell form **7**‐F using 385 nm light, the system underwent a transition to the open‐shell diradical form **7**‐T. Due to the bulky design, the thermal back reaction is suppressed at room temperature with an activation barrier of Δ*G* ^‡^ = 25.7 kcal mol^−1^ at 293 K in solution. Even at 328 K (55 °C), the diradical half‐life time is ca. 4 h. Due to the remarkable stability of diradical **7**‐T, it was successfully isolated via chemical reduction and characterized by EPR, revealing a triplet ground state with 2 *J*/*k*_B_ = 11 K (Δ*E*_ST_ = 22·10^−3^ kcal mol^−1^).

In addition to the photo‐induced switching behavior, installation of the peripheral thioxanthene motif enabled access to a stable dicationic state. Electronic oxidation of both **7**‐F and **7**‐T resulted in the formation of the dicationic species **7** ^2+^, which could be reduced back to **7**‐T, highlighting the unique reversible redox‐switching capability. Among the reported spin‐state switches, the combination of bistability and the paramagnetic ground state of the diradical is unprecedented. Overall, certain overcrowded aromatic enes (OAEs) exhibit very robust spin‐state photoswitching behavior, positioning them as promising molecular scaffolds with demonstrated proof‐of‐concept applications.

Despite one example claiming a photo‐induced back reaction,^[^ ^105^ ^]^ the conversion from open‐shell to closed‐shell form is only realized thermally. There is no rational principle for a photo‐triggered back reaction based on reestablishing strain of the overcrowded double bond. It has been challenging to rationally design a system that forms a paramagnetic species upon irradiation with light.

Empirical analysis of the reported systems suggests a sweet spot of sterically induced strain in the fjord region and flexibility of the peripheral substituent—the example by Feringa might so far have come closest.

## 4 Photochemical Spin‐State Switches

**Figure 6:**
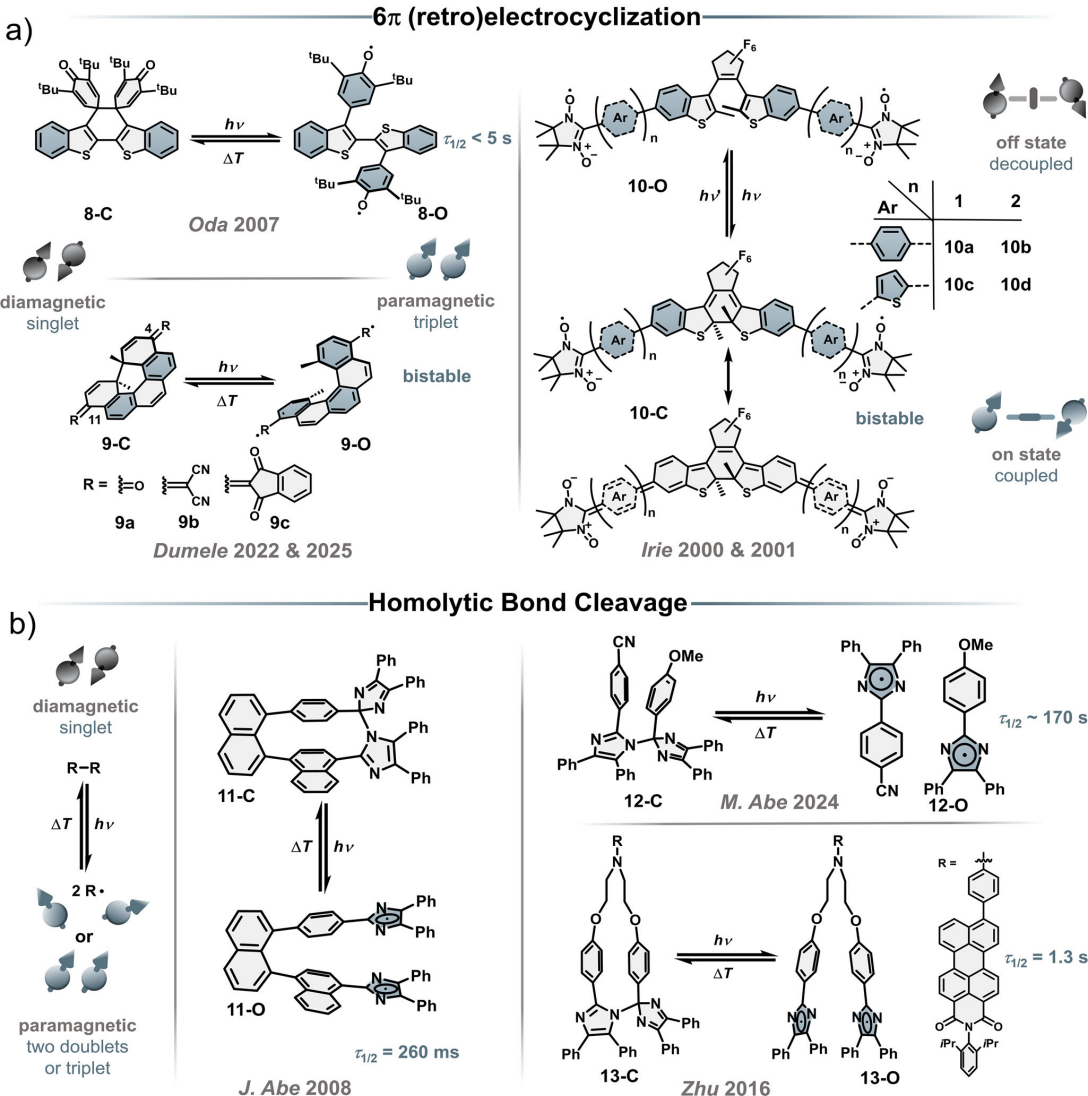
Selected examples of photochemical switches based on a) 6π (retro)electrocyclization and b) homolytic bond cleavage.

**Figure 7:**
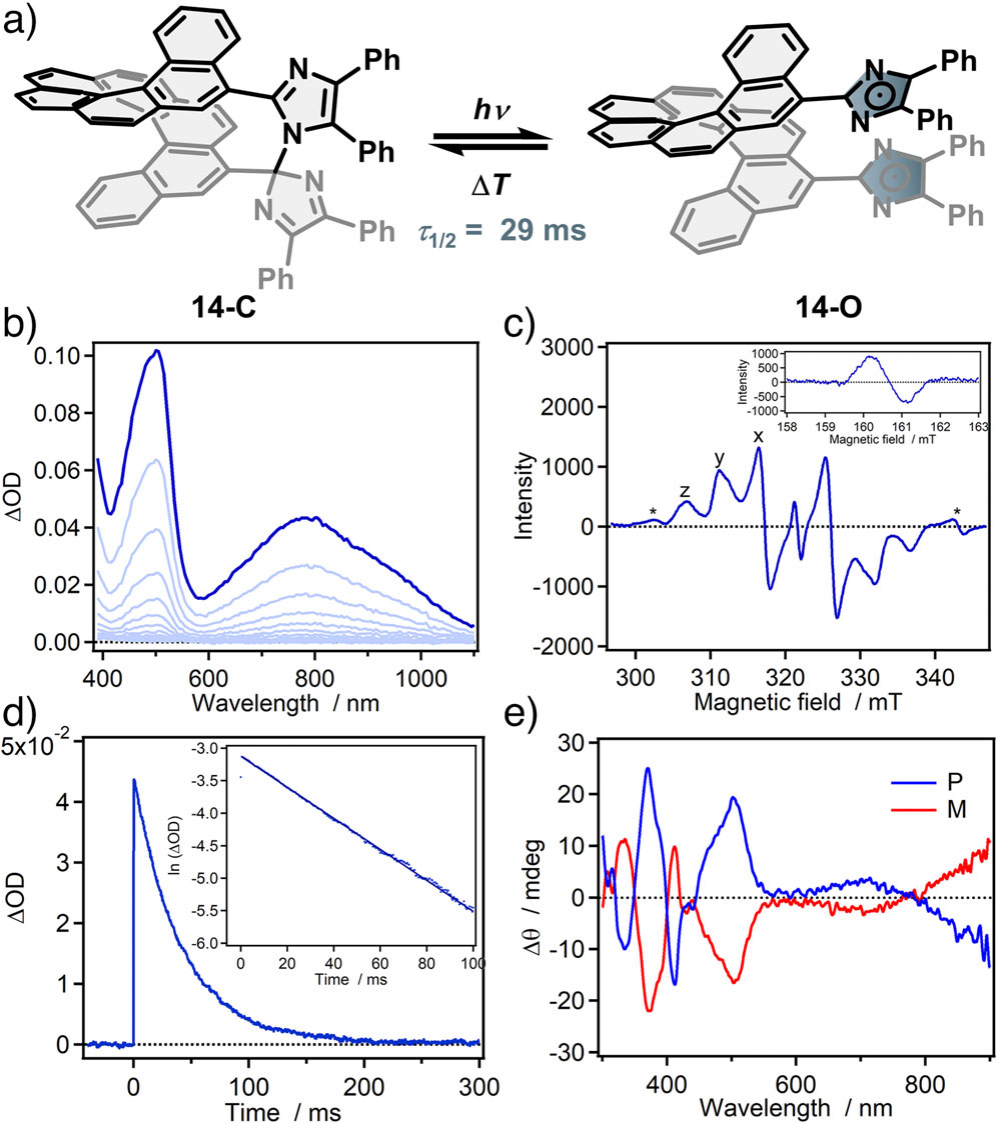
a) Photochemical spin‐state switching of **14**‐C to **14**‐O; b) Transient absorbance spectral change upon irradiation of **14**‐C with 365 nm light; c) EPR spectrum of **14**‐O, inset shows signal at half‐field; d) Time profile of transient absorbance for the thermal back reaction at 800 nm, inset shows first‐order kinetic plot; e) ECD spectra of **14**‐O. Figures were edited and reprinted with permission of J. Abe and under the CC BY 3.0 licence.^[^ ^64^ ^]^

Besides the aforementioned photoconformational spin‐state switching that relies on the release of strain or *cis*–*trans* isomerization, a second class has been established. Here, a photochemical reaction causes a change in connectivity of the molecular scaffold, resulting in a different electronic spin state. This class can be further divided into two subgroups. The molecular scaffold change can occur through a formal pericyclic reaction (**8**–**10**, Figure 6) or by homolytic bond cleavage (**11**–**14**, Figures 6 and 7). To this date the latter solely consists of the vast family of hexaarylbiimidazoles (HABI), with selected examples discussed in this review.^[^ ^58^, ^62^, ^64^, ^111^ ^]^

An early example of photochemical spin‐state switching by 6π retroelectrocyclization was reported by Oda and co‐workers in 2007 with a 3,3′‐bis(2,6‐di‐*tert*‐butylcyclohexadienone) thioindigo **8** that exhibits a weak EPR signal at room temperature, which increases upon irradiation using a Xenon lamp (Figure 6a).^[^ ^112^ ^]^ It is suggested that the Woodward–Hoffmann (WH) rules^[^ ^113^ ^]^ allowed photochemical 6π retroelectrocyclization of **8**‐C leads to the ring‐open diradical **8**‐O, which is stabilized by the gain of aromaticity with two new Clar's sextets.^[^ ^114^ ^]^ The thermal back reaction is formally forbidden according to the WH rules but proceeds regardless due to the radical nature of the starting material. EPR spectroscopy of **8**‐O suggests the presence of a triplet diradical, giving the earliest literature entry of a photochemical spin‐state switch that is reversibly interconvertible between a closed‐shell diamagnetic form **8**‐C and an open‐shell paramagnetic triplet form **8**‐O through a pericyclic reaction. At room temperature in the dark, the EPR signal decays within ∼5 s, returning to its initial state.

Also, utilizing a 6π electrocyclization, but in a helicene scaffold,^[^ ^115^ ^]^ to achieve all‐organic spin‐state switching was introduced by our group (**9**, Figure 6), which is discussed further below.^[^ ^63^ ^]^

The opening and closing reaction of diarylethenes (DAE) has been used to control the communication between two spin centers. Through a photochemical 6π (retro)electrocyclization of DAEs, the reversible interconversion between two different paramagnetic states is achieved by changing the electronic exchange integral *J* (Figure 6a).^[^ ^66^, ^67^, ^68^, ^69^, ^70^ ^]^ Matsuda and Irie reported on different DAE molecules decorated with two nitronyl nitroxide radicals. The persistent radicals are connected to a spin‐coupling core through a spacer moiety, which is essential for the variation of the exchange integral *J*. With a single benzene spacer (**10a**‐O), an increase of the exchange interaction between the two radicals was observed when switching from the open form **10a**‐O to the closed form **10a**‐C (from 2 *J*/*k*_B_ = 1.2·10^−3^ K to 2 *J*/*k*_B_ > 40·10^−3^ K) using 313 nm light.^[^ ^66^, ^68^ ^]^ This observation is a direct result of the increased linear conjugation from the contribution of the quinoidal resonance structure in **10a**‐C. Extending the spacer to biphenyl (**10b**‐O) results in the same effect of an increased coupling in the ring‐closed form **10b**‐C. The increase in *J* is, however, not as drastic because of the reduced quinoidal character, which is significantly diminished with a longer spacer.^[^ ^68^ ^]^ When a thiophene (**10c**‐O) or bithiophene (**10d**‐O) spacer is used, the effects are similar (Table 1).^[^ ^67^ ^]^ Notably, if no spacer is used, the overall trend of an increasing exchange interaction is still measurable; nevertheless, no change in the EPR spectrum is observed due to the strong coupling in both ring‐open and ring‐closed isomers.^[^ ^66^ ^]^

**Table 1:** Comparison of the magnetic exchange integrals *J* for the selected DAEs.^[^ ^66^, ^67^, ^68^ ^]^

	Ring‐open isomer ‐O	Ring‐closed isomer ‐C
Compound	$\frac{2J}{k_{B}}$ (mK)	$\frac{2J}{k_{B}}$ (mK)
**10a**	1.2	>40
**10b**	<0.3	10
**10c**	5.6	>40
**10d**	<0.3	>40

Reversible photochemical spin‐state switching through homolytic bond cleavage has to date only been reported on hexaarylbiimidazoles (HABIs, Figure 6b).^[^ ^58^, ^62^, ^64^, ^74^, ^111^, ^116^, ^117^, ^118^, ^119^, ^120^ ^]^ In general, a C─N bond between two imidazoles is homolytically cleaved upon irradiation, resulting in a change of the spin state from a closed‐shell form to an open‐shell triplet or bis‐doublet state. The newly formed short‐lived paramagnetic system recombines thermally with generally short half‐life times in the range of milliseconds to seconds. Initially discovered in the 1960s by Maeda, a HABI spin‐state switch was reported by J. Abe and co‐workers in 2008 (Figure 6b).^[^ ^111^, ^119^ ^]^ A naphthalene‐bridged imidazole dimer **11**‐C rapidly undergoes intramolecular homolytic bond cleavage. Upon irradiation with 365 nm light, the colorless solution changed to green. Ceasing irradiation readily re‐established the initial state with a half‐life time of *τ*_1/2_ = 260 ms.^[^ ^111^ ^]^ Intermolecular examples of such spin‐state switching with HABIs have been achieved from symmetric and asymmetric monomers, both in a crystal and in solution.^[^ ^62^, ^65^, ^74^, ^120^ ^]^ J. Abe and co‐workers reported the intermolecular spin‐state switching of a HABI consisting of two symmetric monomers in a crystal with both the closed‐shell dimer and open‐shell monomers structurally resolved by single‐crystal X‐ray diffraction.^[^ ^65^, ^120^ ^]^ Interestingly, due to the close proximity of the two radical monomers and hence through‐space radical–radical coupling, the irradiated crystal showed a triplet EPR pattern. ^[^ ^65^, ^120^ ^]^ More recently, M. Abe and co‐workers prepared a number of symmetric and asymmetric HABIs to investigate the impact of the transition‐state aromaticity on radical–radical coupling (Figure 6b for one example).^[^ ^62^ ^]^ Irradiation of HABI **12**‐C, consisting of an electron‐deficient cyano‐substituted and an electron‐rich methoxy‐substituted imidazole, using 365 nm light leads to the formation of two imidazolyl radicals with a doublet EPR signal. Ceasing the irradiation causes recombination of the radicals to the initial state with a remarkably long half‐life time of *τ*_1/2_ ≈ 170 s. This half‐life time is longer than for the symmetric unsubstituted dimer (*τ*_1/2_ ≈ 37 s) but shorter than for the symmetric methoxy dimer (*τ*_1/2_ ≈ 400 s) and significantly shorter than for the symmetric cyano dimer (*τ*_1/2_ ≈ 2260 s).^[^ ^62^ ^]^ The spin‐state switching of HABIs has also found first applications utilizing their fluorescent and magnetic properties.^[^ ^58^, ^118^ ^]^ Perylenemonoimide‐HABI **13**‐C is highly fluorescent in the visible region with a quantum yield of *ϕ* = 0.63 in cyclohexane and NIR fluorescent with a quantum yield of *ϕ* = 0.29 in CH_2_Cl_2_. Irradiation with UV light leads to a near complete quenching of the fluorescence and the generation of an uncoupled radical pair evidenced by a doublet signal in the EPR. In general, intramolecular HABI's result in a through‐space coupled triplet state upon irradiation; **13**‐O, however, shows a doublet signal due to the flexible ethylene bridges between the imidazole moieties and the perylenemonoimide.^[^ ^58^ ^]^ The thermal recombination of the radical pair occurs with a half‐life time of *τ*_1/2_ = 1.3 s.^[^ ^58^ ^]^ HABI **13**‐C/**13**‐O has also been used for super‐resolution imaging with electrospun nanowires.^[^ ^58^ ^]^

Since the 1960s, the group of HABIs has steadily grown with major contributions from J. Abe and co‐workers. Emphasizing this development, we want to further discuss a recent literature report showcasing the structural diversity of HABIs, including characteristic experimental data. Two [7]helicene‐ and [9]helicene‐bridged bisimidazoles were prepared, and their behavior under irradiation was investigated (Figure 7, only [9]helicene HABI shown).^[^ ^64^ ^]^ Characteristic broad and long wavelength bands, reaching into the NIR region, emerged upon irradiation of **14**‐C with 365 nm light. Ceasing irradiation led to the thermal back reaction of **14**‐O regaining the initial spectrum with a half‐life time of *τ*_1/2_ = 29 ms (Figure 7b,d). The EPR spectrum of **14**‐O indicates a triplet diradical with a low symmetry‐related anisotropy of the observed lines (in a frozen toluene solution at 80 K, Figure 7c).^[^ ^64^, ^71^ ^]^ Strong evidence for a triplet state is the presence of a signal at half field (Δ*m*_s_ = 2).^[^ ^64^, ^71^ ^]^ The chiral nature of **14** was also investigated in the open and closed form, with Cotton^[^ ^121^ ^]^ effects reaching into the near‐infrared region for **14**‐O in circular dichroism spectroscopy (Figure 7e).

**Figure 8:**
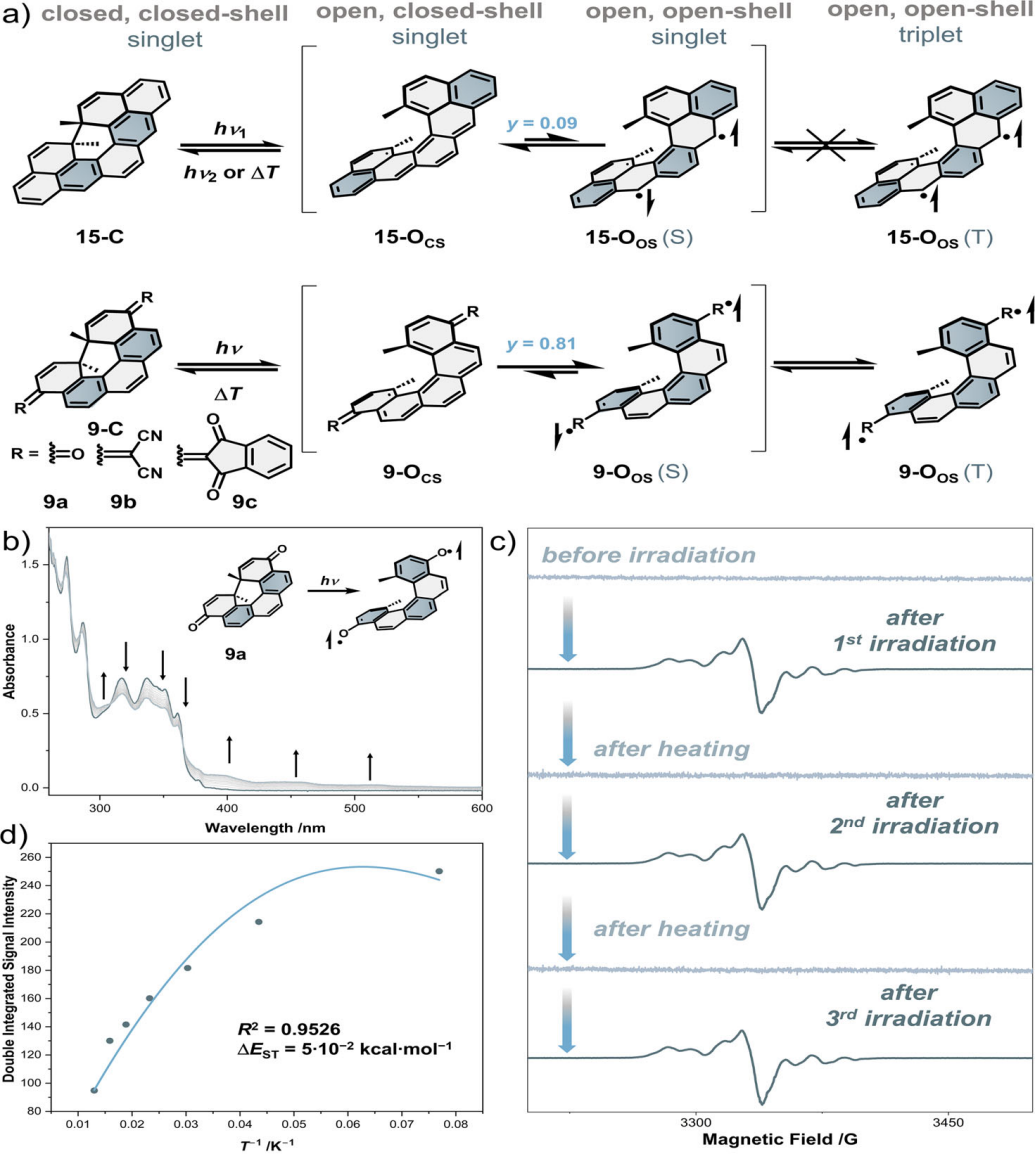
a) Structural comparison of the photoswitching behavior of **9a**‐**c** and **15**; b) UV–vis spectrum of **9a** before and after irradiation with 375 nm light for 10 min in a frozen matrix at 77 K; c) EPR cycles of **9a** before and after irradiation, recorded at 13 K; d) Bleaney–Bowers plot derived from the VT‐EPR.^[^ ^63^ ^]^

Juríček and co‐workers attempted to expand the library of photochemical spin‐state switches with the [5]helicene‐derived dimethylcethrene **15**.^[^ ^122^ ^]^ They intended to use phenalenyl as a radical stabilizing group at a known 6π electrocyclization core earlier reported by Kubo.^[^ ^123^ ^]^ Installing methyl groups in the fjord region, as established in diarylethene photoswitches, allowed for photoreversible switching between a closed and open form (Figure 8).^[^ ^78^, ^115^ ^]^ Despite an efficient photochemical conversion from closed to open form, no EPR signal could be detected. The observation of a thermal back reaction, however, suggests the contribution of an open‐shell singlet form of **15**‐O.

Inspired by Juríček and co‐workers,^[^ ^115^ ^]^ our group reported all‐organic photochemical spin‐state switches with bistable spin states based on a [5]helicene scaffold **9a**‐**c** (Figure 8).^[^ ^63^, ^124^ ^]^ All three derivatives undergo a 6π retroelectrocyclization from their closed forms to the open form under irradiation at 77 K. Whilst diketone **9a**‐C and bis(dicyanomethylidene) **9b**‐C require UV light (365 nm, 405 nm) for the photochemical conversion to their respective open forms (**9a**‐O, **9b**‐O), bis(indanedione) **9c**‐C can be switched with visible light (450 nm). The generation of a paramagnetic triplet species is evidenced by EPR spectroscopy. All three entries can exhibit triplet ground states and can be converted back to their initial diamagnetic closed form thermally at temperatures above 100 K.^[^ ^63^, ^124^ ^]^

Comparing Juríček's dimethylcethrene skeleton **15** with the dimethyl[5]helicene skeleton **9** gives insight into the difference in behaviors under photoirradiation (Figure 8a). Irradiation of the ring‐closed forms (**15**‐C, **9**‐C) initiates a conrotatory 6π retroelectrocyclization to form the ring‐open [5]helicenes. At this point, the number of Clar's sextets determines the ground‐state spin multiplicity. In the case of **9**‐O, the contribution of the closed‐shell (CS) is energetically unfavored due to the complete loss of all Clar's sextets. However, the open‐shell (OS) form **9**‐O_OS_ regains three Clar's sextets, which is a substantial aromatic stabilization. Thus, the CS singlet state of **9**‐O is not energetically stabilized, resulting in a diradical(oid), as indicated by the large diradical index of *y* = 0.81 for **9a**‐O_CS_. In contrast, cethrene **15**‐O_CS_ retains two Clar's sextets, making this resonance structure accessible and the major contributor of the ring‐open form (*y* = 0.09). The observation of a thermal back reaction, but not a photochemical one for **9**‐O, also implies the absence of the ring‐open CS form. According to WH rules, conrotatory photochemical 6π electrocyclization is only allowed for **9**‐O_CS_.^[^ ^113^, ^122^ ^]^ In contrast, dimethylcethrene is readily converted back to its ring‐closed form using visible light. The major mesomeric form for ring‐open dimethyl[5]helicenes **9**‐O is hence an open‐shell electronic configuration **9**‐O_OS_ regaining three Clar's sextets. Paired with a small singlet–triplet energy gap, this overall generates stable triplet diradicals upon irradiation of frozen solutions of **9**‐C. Experimentally, the generation of the ring‐open dimethyl[5]helicene under irradiation can be followed spectroscopically (Figure 8b).^[^ ^63^ ^]^ A broad long‐wavelength absorption band emerges, which can be reversed by warming the matrix above a certain threshold (∼100 K). Evidence for the triplet ground state of open diketone **9a**‐O_OS_(T) is provided by VT‐EPR spectroscopy with an increase of signal intensity at low temperatures (Δ*E*_ST_ = 0.0507 kcal mol^−1^ = 17.7 cm^−1^, Figure 8d).^[^ ^63^ ^]^ The switching cycles can be repeated several times with an alternating irradiation–heating protocol and recording EPR spectra after each stimulus (Figure 8c).

Despite the significant progress in the field of photochemical spin‐state switches, some major challenges remain. Generating an open‐shell paramagnetic state through a light‐induced reaction has been successful; a photochemical reaction back to a diamagnetic state has, however, not yet been achieved, and only thermal back reactions with low activation barriers are possible to date. The low activation barriers result in short half‐life times at elevated temperatures, and bistability can only be obtained at very low temperatures.

## 5 Conclusion and Technological Outlook

The advancement of all‐organic spin‐state photoswitches represents a significant stride in the precise manipulation of molecular spin states using light. By systematically reviewing both photoconformational and photochemical switching mechanisms, this review outlined the design principles, characterization techniques, and structure–function relationships that define this emerging field. From early proof‐of‐concept systems to structurally optimized bistable switches, a broad spectrum of spin dynamics—ranging from subtle magnetic coupling modulations to complete spin‐state transitions—has been demonstrated. Despite these achievements, considerable challenges remain. The design of reversibly switchable systems that operate under ambient conditions, especially those achieving photoinduced spin‐state interconversion in both directions, is still underdeveloped. Most current systems require a thermal back‐conversion or operate at cryogenic temperatures to maintain bistability, limiting practical applications. Furthermore, while UV–vis and EPR spectroscopy remain cornerstones of characterization, their limited resolution in distinguishing nuanced spin interactions and molecular structure determination underscores the need for improved analytical methods.

From a technological perspective, the integration of organic spin‐state switches into functional devices opens new frontiers in spintronics,^[^ ^125^ ^]^ quantum information processing, and high‐resolution magnetic sensing.^[^ ^9^, ^10^, ^126^ ^]^ The ability to reversibly and selectively control spin populations via light, without relying on metal centers, paves the way for lightweight, nontoxic, and structurally tunable materials. Future work will benefit from cross‐disciplinary approaches—merging synthetic organic chemistry, materials science, and theoretical modelling—to design more robust, fast‐responding, and thermally stable systems. Particularly promising are hybrid systems that combine photochromic scaffolds with persistent radicals or redox‐active motifs, enabling multifunctional behavior such as magneto‐optical switching, data storage, or logic operations at the molecular scale. Additionally, leveraging machine learning for property prediction and the rational design of new photoswitch architectures could significantly accelerate progress.

In an especially exciting development, photogenerated diradical species derived from spin‐state photoswitches are increasingly recognized as viable candidates for molecular qubits. These species could form spin‐entangled radical pairs with long‐lived coherence times and spectrally addressable features that may satisfy some of the DiVincenzo criteria for quantum computing.^[^ ^127^, ^128^ ^]^ Recent demonstrations of light‐controlled quantum gate operations in such systems—including coherent manipulation of singlet and triplet spin states—suggest that organic molecules could serve as scalable, optically addressable units in quantum information architectures, with optical readout abilities.^[^ ^129^, ^130^, ^131^, ^132^, ^133^, ^134^ ^]^ As chemical synthesis offers atomically precise control over spin environments, organic spin‐state photoswitches may one day underpin robust, room‐temperature quantum processors or memory devices.

In conclusion, organic spin‐state photoswitches offer an exciting molecular platform for controllable spin manipulation. With continued innovation in molecular design and methodological tools, these systems are poised to make substantive contributions to the next generation of molecular electronics, spin‐based computing, and quantum photonic technologies.

## Supporting Information

The authors have cited additional references within the Supporting Information.^[^ ^17^, ^53^, ^62^, ^83^, ^87^, ^88^, ^89^, ^91^, ^92^, ^93^, ^94^, ^95^, ^96^, ^97^, ^98^, ^135^, ^136^, ^137^, ^138^, ^139^, ^140^, ^141^ ^]^

## Conflict of Interests

The authors declare no conflict of interest.

## Supporting information



Supporting Information

## Data Availability

The data that support the findings of this study are available in the Supporting Information of this article.
