# Supplementary material for: Organic Spin‐State Photoswitches
Source: Angew Chem Int Ed Engl. 2025 Aug 22;64(38):e202512691. doi: 10.1002/anie.202512691 (PMC12435443; doi:10.1002/anie.202512691)
Supplement: Supplementary file 1 — Supporting Information [file ANIE-64-e202512691-s001.pdf]

Supporting Information

for

Organic Spin-State Photoswitches

Takuma Miyamura,†[a,b] Joël Schlecht,†[a,b] and Oliver Dumele*[a,b]

[a] Department of Organic Chemistry, Albert-Ludwigs-Universität Freiburg, Alberstrasse 21, 79104 Freiburg, Germany

[b] Department of Chemistry, University of Cologne, Greinstrasse 4, 50939 Cologne, Germany

E-Mail: odumele@uni-koeln.de

†T.M. and J.S. contributed equally to this work and either has the right to list himself first in bibliographic documents. All authors have given approval to the final version of the manuscript.

Table of Contents

S1. Computational Methods 3

S1.1 Geometry optimization of singlet diradical(oid)s using broken-symmetry (BS)-DFT 3

S1.2 Geometry optimization of triplet state form using unrestricted DFT 4

S1.3 Geometry optimization of open-shell singlet state using BS-DFT 5

S1.4 Diradical Index y_0 7

S1.4 Prediction of EPR 10

S1.5 Aromaticity Computations 12

S1.6 Atomic Coordinates of Geometry-Optimized Structures 12

S2. References 16

S1. Computational Methods

This Supporting Information aims to offer a practical guide for organic chemists on performing and interpreting common computational procedures relevant to all-organic spin-state photoswitches. The content includes protocols for geometry optimization of open-shell organic molecules using density functional theory (DFT), along single-point calculations useful for predicting key physical properties. To facilitate reproducibility and clarity, several examples of actual input files and output data are provided throughout.

Quantum chemical calculations in the gas phase were performed using the Gaussian 16 Revision C.01 package[1] on the JUSTUS 2 high-performance computing cluster by the state of Baden-Württemberg. All the optimized structures were verified as true minima by confirming the absence of imaginary vibrational frequencies. Single-point calculations were conducted based on these DFT-optimized geometries. Natural orbital occupation numbers were computed using the CASSCF methods implemented in Gaussian 16, with systematic expansion of both the basis set and the active space, to finally calculate at CASSCF(12,12)/6-31G(d,p) level of theory. Zero-Field Splitting (ZFS) parameters were obtained using the ORCA 5.0.3 software package,[2] at restricted open-shell (RO) BP/EPR-II level of theory with automatic auxiliary basis sets (AutoAux).

S1.1 Geometry optimization of singlet diradical(oid)s using broken-symmetry (BS)-DFT

To optimize the geometry of singlet diradical(oid) systems, it is generally advisable to begin with the geometry optimization of the corresponding triplet state. The resulting triplet geometry can then serve as the starting point for the singlet diradical(oid) optimization using broken -symmetry (BS)-DFT (**Figure S1**). It is important to note, however, that diradical(oid) systems have multi-reference character, rendering standard DFT approaches potentially unreliable, and therefore, not recommended. For a detailed and practical protocols for DFT calculations in general, see Ref.[3]

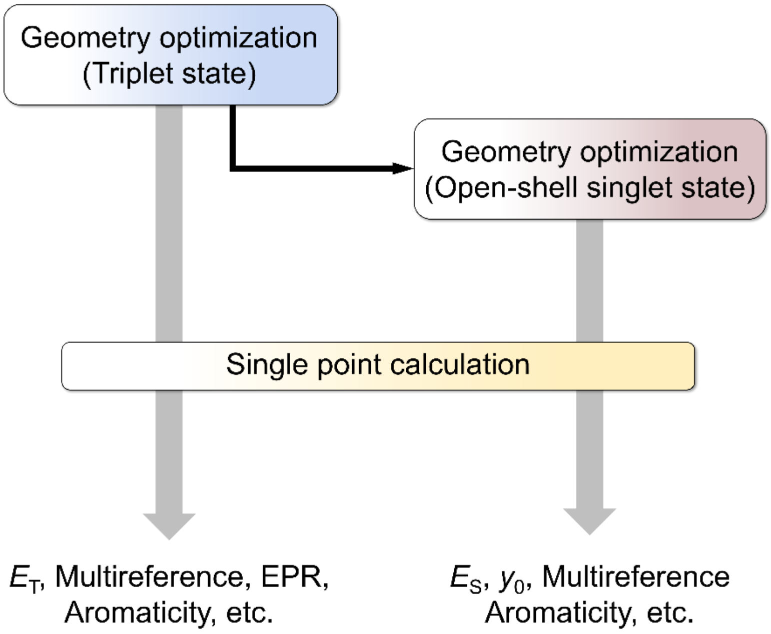

Figure S1. Conceptual flowchart of computing open-shell organic systems using DFT.

The spin-corrected singlet–triplet energy gaps (ΔE_{ST}^{SC}) can be estimated by using the following equation:[4]

$$\Delta E_{ST}^{SC} = \Delta E_{ST} \frac{< S^2 >_T}{< S^2 >_T - < S^2 >_{BS}}$$

Here, ΔE_{ST} represents the computed singlet-triplet energy gap derived from DFT, while $< S^2 >_T$ and $< S^2 >_{BS}$ are the $< S^2 >$ values corresponding to the triplet and BS open-singlet states, respectively. Geometry optimization of triplet form. Gaussian 16 Revision C.01 package was used for the geometry optimizations and a single-point CASSCF calculation.[1]

S1.2 Geometry optimization of triplet state form using unrestricted DFT

In general, an initial optimization of the triplet diradical form is recommended to obtain the open-shell structure of the system. The Gaussian input file for the geometry optimization of simplified Tschitschibabin's hydrocarbon is given below.[5]

Sample Input File

```
%mem=10GB
%nprocshared=11
%chk=simplified-tschitschibabin_opt_freq_triplet.chk
#p opt freq=noraman ub3lyp empiricaldispersion=gd3bj def2tzvp

Optimization of the triplet form

0 3
 C                  -7.78800000    0.06200000   -0.10800000
 C                  -7.10400000   -1.06300000   -0.35400000
 C                  -5.76200000   -1.07600000   -0.31000000
 C                  -5.01200000    0.01200000   -0.02200000
 C                  -5.73900000    1.12800000    0.22000000
 C                  -7.08100000    1.16100000    0.18100000
 C                  -3.65000000   -0.01200000    0.02100000
 C                  -2.90000000    1.07600000    0.31200000
 C                  -1.55700000    1.06300000    0.35600000
 C                  -0.87300000   -0.06200000    0.10700000
 C                  -1.58000000   -1.16000000   -0.18400000
 C                  -2.92200000   -1.12600000   -0.22400000
 C                   0.62900000   -0.10700000    0.15000000
 C                  -9.29000000    0.10700000   -0.14900000
 H                  -9.73800000   -0.57200000    0.61100000
 H                  -9.67700000   -0.15400000   -1.16000000
 H                   1.07700000    0.57800000   -0.60500000
 H                   1.01500000    0.14500000    1.16300000
 H                  -7.65900000   -1.98600000   -0.59500000
 H                  -5.33000000   -2.06300000   -0.53100000
 H                  -5.28700000    2.09900000    0.46800000
 H                  -7.61900000    2.10200000    0.38700000
 H                  -3.33200000    2.06200000    0.53600000
 H                  -1.00200000    1.98400000    0.60000000
 H                  -1.04200000   -2.10100000   -0.39400000
 H                  -3.37400000   -2.09700000   -0.47600000
```

S1.3 Geometry optimization of open-shell singlet state using BS-DFT

Here, the keyword “guess=mix” is used to mix the highest occupied molecular orbital (HOMO) and the lowest unoccupied molecular orbital (LUMO) in the initial guess of the wavefunction. Additionally, “guess=always” is specified enforce HOMO-LUMO mixing at each optimization step, preventing the wavefunction from converging to closed-shell

configuration. As the initial geometry for the singlet optimization, the geometry of pre-optimized triplet diradical is used, which was obtained from the former Gaussian calculation.

Sample Input File

```
%mem=10GB
%nprocshared=11
%chk=tschitschibabin-open-singlet.chk
#p opt freq=noraman ub3lyp empiricaldispersion=gd3bj guess=(mix,always)
def2tzvp

Optimization of the open-shell form using BS-DFT

0 1
 C                 -3.58455100   -0.00001300    0.00002100
 C                 -2.84050500   -1.14255400    0.39423600
 C                 -1.46300800   -1.13653900    0.39155700
 C                 -0.73756600    0.00005900    0.00006000
 C                 -1.46306600    1.13660400   -0.39149300
 C                 -2.84058300    1.14258000   -0.39414900
 C                  0.73756800    0.00006000   -0.00004500
 C                  1.46303500    1.13660400    0.39147600
 C                  2.84061100    1.14254100    0.39409400
 C                  3.58451700   -0.00000500   -0.00002200
 C                  2.84053000   -1.14252100   -0.39418800
 C                  1.46298600   -1.13655800   -0.39153100
 C                  4.98616600   -0.00009900    0.00006300
 C                 -4.98611600   -0.00010600   -0.00008300
 H                 -5.54332200    0.87639900   -0.29940300
 H                 -5.54326900   -0.87661700    0.29931600
 H                  5.54335000    0.87616400    0.30012400
 H                  5.54327500   -0.87639900   -0.30002800
 H                 -3.37494200   -2.03010100    0.70980300
 H                 -0.92732100   -2.01767000    0.72029800
 H                 -0.92743800    2.01772800   -0.72030700
 H                 -3.37504600    2.03012200   -0.70967900
 H                  0.92743900    2.01771900    0.72033700
 H                  3.37497500    2.03013300    0.70965000
 H                  3.37487300   -2.03011500   -0.70978600
 H                  0.92732100   -2.01767400   -0.72030500
```

S1.4 Diradical Index y_0

The contribution of its open-shell resonance structure to the singlet state of diradical(oid) can be described with diradical index y_0 (**Figure S2**). It should not be confused with a method to quantify the triplet state or the paramagnetism, but this index is only for the singlet state of diradical(oid)s. The index y_0 range between 0 and 1, where y_0 = 1 indicates a purely open-shell system and y_0 = 0, a complete closed-shell system.

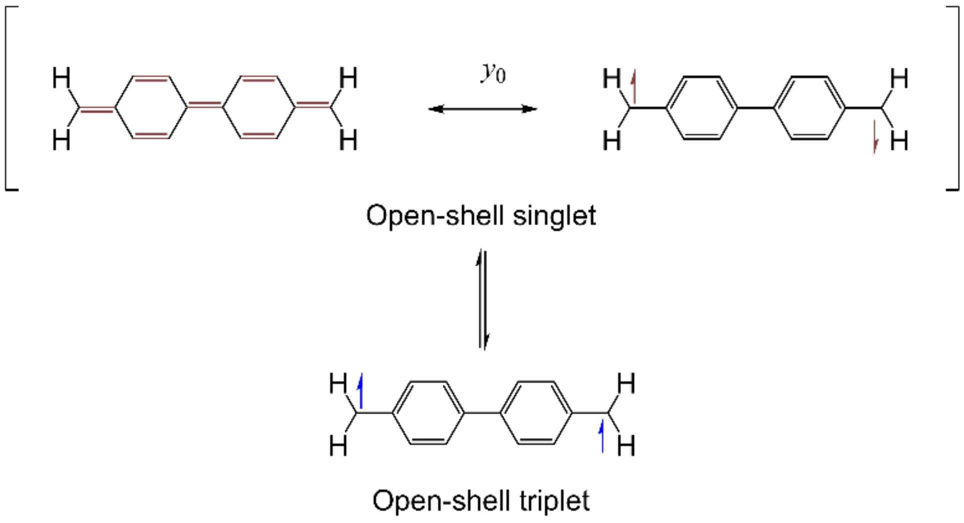

Figure S2. Conceptual scheme of diradical index

In diradical(oid) systems, mixing of HOMO and LUMO occurs resulting in characters between closed-shell and open-shell. The singlet open-shell character can be evaluated quantum chemically by using a diradical index y_0 following this equation proposed by Yamaguchi:[6]

$$y_0 = 1 - \frac{2T}{1+T}, \qquad T = \frac{n_{\mathrm{HONO}} - n_{\mathrm{LUNO}}}{2}$$

where n_{HONO} and n_{LUNO} are the occupancy of the highest occupied natural orbital (HONO) and the lowest unoccupied natural orbital (LUNO), respectively. As the distribution of electrons between n_{HONO} and n_{LUNO} becomes closer to 1 to 1, the degree of diradical character strengthens where y_0 approaches 1.

Sample Input File

```
%mem=10GB
%nprocshared=11
%chk=tschitschibabin-open-singlet_casscf_pop-no.chk
#p rhf/sto-3g

CASSCF calculation using open-singlet geometry

0 1
 C                 -3.59573800   -0.00000100    0.00000500
 C                 -2.82640100   -1.22065200   -0.00003000
 C                 -1.47309200   -1.21561800   -0.00002900
 C                 -0.70424500    0.00000100    0.00000200
 C                 -1.47309300    1.21561900    0.00003600
 C                 -2.82640200    1.22065100    0.00004000
 C                  0.70424500    0.00000100   -0.00000100
 C                  1.47309300    1.21561900    0.00002100
 C                  2.82640200    1.22065100    0.00001700
 C                  3.59573800    0.00000000   -0.00000500
 C                  2.82640100   -1.22065200   -0.00002700
 C                  1.47309200   -1.21561800   -0.00002800
 C                  4.95518300   -0.00000100   -0.00000600
 C                 -4.95518300   -0.00000100    0.00000600
 H                 -5.51664700    0.92447600    0.00003300
 H                 -5.51664600   -0.92448000   -0.00002200
 H                  5.51664700    0.92447600    0.00001000
 H                  5.51664600   -0.92448000   -0.00002100
 H                 -3.36244900   -2.16193800   -0.00005800
 H                 -0.96160500   -2.16607700   -0.00005600
 H                 -0.96160700    2.16607900    0.00006500
 H                 -3.36245100    2.16193700    0.00007100
 H                  0.96160700    2.16607900    0.00003700
 H                  3.36245100    2.16193700    0.00003000
 H                  3.36244900   -2.16193800   -0.00004300
 H                  0.96160500   -2.16607700   -0.00004500


--link1--
%mem=10GB
%nprocshared=11
%chk=tschitschibabin-open-singlet_casscf_pop-no.chk
#p casscf(2,2)/sto-3g scf(maxcycle=1000) guess=read pop=no
geom=allcheck

--link1--
%mem=10GB
%nprocshared=11
%chk=tschitschibabin-open-singlet_casscf_pop-no.chk
#p casscf(2,2)/4-31g scf(maxcycle=1000) guess=read pop=no geom=allcheck
```

```
--link1--
%mem=10GB
%nprocshared=11
%chk=tschitschibabin-open-singlet_casscf_pop-no.chk
#p casscf(2,2)/6-31g(d) scf(maxcycle=1000) guess=read pop=no
geom=allcheck

--link1--
%mem=10GB
%nprocshared=11
%chk=tschitschibabin-open-singlet_casscf_pop-no.chk
#p casscf(2,2)/6-31g(d,p) scf(maxcycle=1000) guess=read pop=no
geom=allcheck

--link1--
%mem=10GB
%nprocshared=11
%chk=tschitschibabin-open-singlet_casscf_pop-no.chk
#p casscf(4,4)/6-31g(d,p) scf(maxcycle=1000) guess=read pop=no
geom=allcheck

--link1--
%mem=10GB
%nprocshared=11
%chk=tschitschibabin-open-singlet_casscf_pop-no.chk
#p casscf(6,6)/6-31g(d,p) scf(maxcycle=1000) guess=read pop=no
geom=allcheck

--link1--
%mem=10GB
%nprocshared=11
%chk=tschitschibabin-open-singlet_casscf_pop-no.chk
#p casscf(8,8)/6-31g(d,p) scf(maxcycle=1000) guess=read pop=no
geom=allcheck

--link1--
%mem=10GB
%nprocshared=11
%chk=tschitschibabin-open-singlet_casscf_pop-no.chk
#p casscf(10,10)/6-31g(d,p) scf(maxcycle=1000) guess=read pop=no
geom=allcheck

--link1--
%mem=10GB
%nprocshared=11
%chk=tschitschibabin-open-singlet_casscf_pop-no.chk
#p casscf(12,12)/6-31g(d,p) scf(maxcycle=1000) guess=read pop=no
geom=allcheck
```

The output file contains natural orbital coefficient eigenvalues. Since the 48th and the 49th orbitals are the HONO and LUNO of the molecule, respectively, y_0 is derived as 0.15 at CASSCF(12,12)/6-31G(d,p)//BS-B3LYP-D3(BJ)/def2TZVP level of theory.

S1.4 Simulation of EPR Spectra

In some cases, predicting the EPR spectrum helps the characterization of the paramagnetic form of the switch. Specifically for organic spin-state switches, zero-field splitting (ZFS) prediction is one of the powerful methods for characterization of systems with $S > 1/2$, together with experimental EPR spectral data. The comparison between the computationally predicted D values and E/D ratios with their experimentally obtained counterparts not only provides further validation of spin-state switching phenomena but also offers valuable insights into the structural and electronic features of di(bi)radical state (**Figure S3**).[7] Such parameters can be calculated by, for example, the ORCA software,[2] and the spectral simulation can be done by using software such as Easyspin.[8]

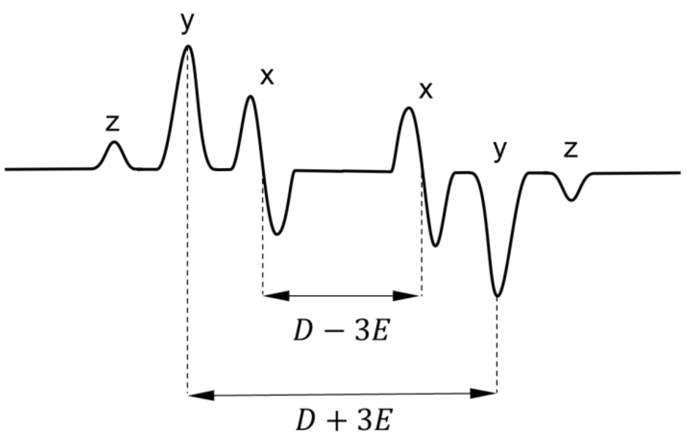

Figure S3. Parameters (*E* and *D* values) which can be extracted from EPR spectrum

Here, we provide one example of ZFS calculation input file for ORCA using the triplet state of the simplified Tschitschibabin's hydrocarbon.

Sample Input File

```
! ROKS bp EPR-II autoaux rijcosx

%maxcore 3000
%pal nprocs 3 end

* xyz 0 3
 C                 -3.58455100   -0.00001300    0.00002100
 C                 -2.84050500   -1.14255400    0.39423600
 C                 -1.46300800   -1.13653900    0.39155700
 C                 -0.73756600    0.00005900    0.00006000
 C                 -1.46306600    1.13660400   -0.39149300
 C                 -2.84058300    1.14258000   -0.39414900
 C                  0.73756800    0.00006000   -0.00004500
 C                  1.46303500    1.13660400    0.39147600
 C                  2.84061100    1.14254100    0.39409400
 C                  3.58451700   -0.00000500   -0.00002200
 C                  2.84053000   -1.14252100   -0.39418800
 C                  1.46298600   -1.13655800   -0.39153100
 C                  4.98616600   -0.00009900    0.00006300
 C                 -4.98611600   -0.00010600   -0.00008300
 H                 -5.54332200    0.87639900   -0.29940300
 H                 -5.54326900   -0.87661700    0.29931600
 H                  5.54335000    0.87616400    0.30012400
 H                  5.54327500   -0.87639900   -0.30002800
 H                 -3.37494200   -2.03010100    0.70980300
 H                 -0.92732100   -2.01767000    0.72029800
 H                 -0.92743800    2.01772800   -0.72030700
 H                 -3.37504600    2.03012200   -0.70967900
 H                  0.92743900    2.01771900    0.72033700
 H                  3.37497500    2.03013300    0.70965000
 H                  3.37487300   -2.03011500   -0.70978600
 H                  0.92732100   -2.01767400   -0.72030500
*
%eprnmr
 dtensor ss
 dss direct
end
```

For the triplet state of simplified Tschitschibabin's hydrocarbon, predicted ZFS parameters are:

```
D   =    -0.020639  cm**-1
E/D =     0.010622
```

We also direct interested reader to the recent literature on this topic, including simulations of time-resolved EPR spectroscopy.[9]

S1.5 Aromaticity Computations

Because many switches possess an aromatic backbone to facilitate switching behavior, their functionality often originates from the gain and loss of aromaticity based on *Clar's* sextet rule. Thereby, the analysis of aromaticity is crucial. In this context, nucleus-independent chemical shifts,[10] current density,[11] electron density of delocalized bonds,[12] anisotropy of the induced current,[13] are well-known analyzing methods to visualize and qualitatively make a comparison of degree of aromaticity between the metastable states of switches. A wide range of software is available to evaluate the aromaticity and we refer to the cited literature above.

S1.6 Atomic Coordinates of Geometry-Optimized Structures Discussed in This Review

Simplified Tschitschibabin's hydrocarbon triplet

uB3LYP-D3(BJ)/def2TZVP

Charge = 0, Multiplicity = 3 $<S^2>$ = 2.0469

Number of imaginary frequencies = 0

Sum of electronic and zero-point energies = -540.680394

Sum of electronic and thermal energies = -540.668774

Sum of electronic and enthalpy energies = -540.667829

Sum of electronic and thermal free energies = -540.719100

C	-3.58455100	-0.00001300	0.00002100
C	-2.84050500	-1.14255400	0.39423600
C	-1.46300800	-1.13653900	0.39155700
C	-0.73756600	0.00005900	0.00006000
C	-1.46306600	1.13660400	-0.39149300
C	-2.84058300	1.14258000	-0.39414900
C	0.73756800	0.00006000	-0.00004500
C	1.46303500	1.13660400	0.39147600
C	2.84061100	1.14254100	0.39409400
C	3.58451700	-0.00000500	-0.00002200
C	2.84053000	-1.14252100	-0.39418800
C	1.46298600	-1.13655800	-0.39153100
C	4.98616600	-0.00009900	0.00006300

C	-4.98611600	-0.00010600	-0.00008300
H	-5.54332200	0.87639900	-0.29940300
H	-5.54326900	-0.87661700	0.29931600
H	5.54335000	0.87616400	0.30012400
H	5.54327500	-0.87639900	-0.30002800
H	-3.37494200	-2.03010100	0.70980300
H	-0.92732100	-2.01767000	0.72029800
H	-0.92743800	2.01772800	-0.72030700
H	-3.37504600	2.03012200	-0.70967900
H	0.92743900	2.01771900	0.72033700
H	3.37497500	2.03013300	0.70965000
H	3.37487300	-2.03011500	-0.70978600
H	0.92732100	-2.01767400	-0.72030500

Simplified Tschitschibabin’s hydrocarbon open-singlet

BS-uB3LYP-D3(BJ)/def2TZVP

Charge = 0, Multiplicity = 1 $\langle S^2 \rangle = 0.2396$

Number of imaginary frequencies = 0

Sum of electronic and zero-point energies = -540.692503

Sum of electronic and thermal energies = -540.680925

Sum of electronic and enthalpy energies = -540.679981

Sum of electronic and thermal free energies = -540.730921

C	-3.59573800	-0.00000100	0.00000500
C	-2.82640100	-1.22065200	-0.00003000
C	-1.47309200	-1.21561800	-0.00002900
C	-0.70424500	0.00000100	0.00000200
C	-1.47309300	1.21561900	0.00003600
C	-2.82640200	1.22065100	0.00004000
C	0.70424500	0.00000100	-0.00000100
C	1.47309300	1.21561900	0.00002100
C	2.82640200	1.22065100	0.00001700
C	3.59573800	0.00000000	-0.00000500
C	2.82640100	-1.22065200	-0.00002700
C	1.47309200	-1.21561800	-0.00002800
C	4.95518300	-0.00000100	-0.00000600
C	-4.95518300	-0.00000100	0.00000600
H	-5.51664700	0.92447600	0.00003300
H	-5.51664600	-0.92448000	-0.00002200
H	5.51664700	0.92447600	0.00001000
H	5.51664600	-0.92448000	-0.00002100
H	-3.36244900	-2.16193800	-0.00005800
H	-0.96160500	-2.16607700	-0.00005600
H	-0.96160700	2.16607900	0.00006500

H	-3.36245100	2.16193700	0.00007100
H	0.96160700	2.16607900	0.00003700
H	3.36245100	2.16193700	0.00003000
H	3.36244900	-2.16193800	-0.00004300
H	0.96160500	-2.16607700	-0.00004500

9a Ring-open open-shell singlet (9a-O_{OS})

BS-uCAM-B3LYP-D3(BJ)/def2SVP

Charge = 0, Multiplicity = 1 $\langle S^2 \rangle$ = 1.2024

Number of imaginary frequencies = 0

Sum of electronic and zero-point energies = -1072.976047

Sum of electronic and thermal energies = -1072.956801

Sum of electronic and enthalpy energies = -1072.955857

Sum of electronic and thermal free energies = -1073.022036

C	1.39745100	2.32291400	-0.17867100
C	-1.39741700	2.32292500	0.17866700
C	0.66646500	3.54948600	-0.13432200
C	0.71902500	1.09813500	0.04624800
C	-0.71900600	1.09814200	-0.04626900
C	-0.66642200	3.54949100	0.13431700
H	1.21236300	4.48605800	-0.26485600
H	-1.21231100	4.48606700	0.26486100
C	-1.51306100	-0.06903200	-0.36396700
C	-2.79299900	2.31557400	0.43548700
H	-3.29119600	3.26255500	0.65385000
C	-3.49280500	1.13684800	0.44477700
H	-4.55080200	1.08917300	0.70428800
C	-2.87575400	-0.05519100	0.01817100
C	-3.71094800	-1.27220500	-0.06329300
C	-3.09024800	-2.43696200	-0.66141300
C	-1.04168300	-1.18366700	-1.14011400
C	-1.83912800	-2.35068200	-1.21135000
C	1.51306500	-0.06904600	0.36395500
C	2.87576800	-0.05521300	-0.01814800
C	2.79303700	2.31555200	-0.43546800
H	3.29124500	3.26253000	-0.65381800
C	3.49283600	1.13682200	-0.44474100
H	4.55083800	1.08914000	-0.70422800
C	1.04165300	-1.18368400	1.14007400
C	1.83908900	-2.35070400	1.21133200
C	3.71095200	-1.27223200	0.06333800
C	3.09022600	-2.43698800	0.66143600
C	0.16950300	-1.14598700	-2.02970600

H	-0.09926600	-1.56264700	-3.01197300
H	0.99221500	-1.76117500	-1.63628700
H	0.55331200	-0.13262100	-2.18208300
C	-0.16958000	-1.14600800	2.02960400
H	-0.99227300	-1.76118900	1.63613600
H	-0.55339300	-0.13264100	2.18196800
H	0.09913600	-1.56267600	3.01188300
H	-1.43751400	-3.20380500	-1.76479400
H	1.43744900	-3.20382900	1.76475400
H	-3.69161300	-3.34554700	-0.71805800
H	3.69158400	-3.34557700	0.71809400
O	4.87084100	-1.28586400	-0.34937400
O	-4.87082600	-1.28582900	0.34944900

15c Ring-open open-shell singlet (15c-O$_{OS}$)

BS-uCAM-B3LYP-D3(BJ)/def2SVP

Charge = 0, Multiplicity = 1 $\langle S^2 \rangle$ = 0.7925

Number of imaginary frequencies = 0

Sum of electronic and zero-point energies = -1153.435215

Sum of electronic and thermal energies = -1153.414526

Sum of electronic and enthalpy energies = -1153.413582

Sum of electronic and thermal free energies = -1153.482305

C	0.67328100	3.49416200	-0.05870900
C	-0.67329000	3.49416000	0.05871400
C	-1.41056000	2.25790500	0.04616600
C	-0.69349400	1.01305400	-0.12413600
C	0.69349000	1.01305600	0.12414800
C	1.41055300	2.25790800	-0.04616000
C	-2.77564100	2.24108200	0.25634500
C	-3.52128300	1.02733800	0.25401200
C	-2.87420800	-0.16706900	-0.19881800
C	-1.48014900	-0.15596400	-0.52986900
C	1.48014900	-0.15595900	0.52987800
C	2.87420500	-0.16706400	0.19881300
C	3.52127500	1.02734300	-0.25402200
C	2.77563300	2.24108700	-0.25634800
C	0.96727200	-1.22608500	1.28783300
C	1.77039800	-2.37340300	1.47002800
C	3.05080600	-2.46489600	0.98336000
C	3.65588300	-1.34052600	0.37473200
C	-3.65588400	-1.34053100	-0.37474500
C	-3.05080000	-2.46490000	-0.98336800
C	-1.77038600	-2.37340800	-1.47002000

C	-0.96726100	-1.22609100	-1.28781400
C	5.02030200	-1.34069300	-0.01444400
C	5.61352300	-0.19489200	-0.50195800
C	4.87659500	0.99106700	-0.59734600
C	-4.87660500	0.99106100	0.59732400
C	-5.61353300	-0.19489800	0.50192900
C	-5.02030600	-1.34069800	0.01441800
C	0.36321800	-1.22226000	-1.99029400
C	-0.36319300	-1.22224800	1.99034100
H	1.23371800	4.42888500	-0.12871400
H	-1.23372900	4.42888300	0.12871600
H	-3.29731700	3.17935900	0.45874900
H	3.29730600	3.17936400	-0.45875700
H	1.34627800	-3.21272900	2.02667400
H	3.63423200	-3.37823000	1.11857300
H	-3.63422500	-3.37823400	-1.11858800
H	-1.34625900	-3.21273500	-2.02665900
H	5.59838300	-2.25996200	0.10196000
H	6.66704600	-0.20285800	-0.78853400
H	5.36080500	1.91108700	-0.93204500
H	-5.36081900	1.91108000	0.93201900
H	-6.66705800	-0.20286500	0.78849500
H	-5.59838600	-2.25996700	-0.10199100
H	0.24740100	-1.68580100	-2.98108500
H	1.12269400	-1.80210300	-1.44664900
H	0.76056700	-0.21057300	-2.12814200
H	-0.76054000	-0.21056000	2.12818700
H	-0.24735400	-1.68577900	2.98113400
H	-1.12268000	-1.80209900	1.44671800

S2. References

[1] M. J. Frisch, G. W. Trucks, H. B. Schlegel, G. E. Scuseria, M. A. Robb, J. R. Cheeseman, G. Scalmani, V. Barone, G. A. Petersson, H. Nakatsuji, X. Li, M. Caricato, A. V. Marenich, J. Bloino, B. G. Janesko, R. Gomperts, B. Mennucci, H. P. Hratchian, J. V. Ortiz, A. F. Izmaylov, J. L. Sonnenberg, D. Williams-Young, F. Ding, F. Lipparini, F. Egidi, J. Goings, B. Peng, A. Petrone, T. Henderson, D. Ranasinghe, V. G. Zakrzewski, J. Gao, N. Rega, G. Zheng, W. Liang, M. Hada, M. Ehara, K. Toyota, R. Fukuda, J. Hasegawa, M. Ishida, T. Nakajima, Y. Honda, O. Kitao, H. Nakai, T. Vreven, K. Throssell, J. A. Montgomery, Jr., J. E. Peralta, F. Ogliaro, M. J. Bearpark, J. J. Heyd, E. N. Brothers, K. N. Kudin, V. N. Staroverov, T. A. Keith, R. Kobayashi, J. Normand, K. Raghavachari, A. P. Rendell, J. C. Burant, S. S. Iyengar, J. Tomasi, M. Cossi, J. M. Millam, M. Klene, C. Adamo, R. Cammi, J. W. Ochterski, R. L. Martin, K. Morokuma, O. Farkas, J. B. Foresman, and D. J. Fox, *Gaussian 16, Revision C.01*, Wallingford CT, **2016**.

[2] F. Neese, *Wiley Interdiscip. Rev.: Comput. Mol. Sci.* **2012**, *2*, 73.

[3] M. Bursch, J.-M. Mewes, A. Hansen, S. Grimme, *Angew. Chem. Int. Ed.* **2022**, *61*, e202205735.

[4] K. Yamaguchi, F. Jensen, A. Dorigo, K. N. Houk, *Chem. Phys. Lett.* **1988**, *149*, 537.

[5] A. E. Tschitschibabin, *Ber. Dtsch. Chem. Ges.* **1907**, *40*, 1810.

[6] K. Yamaguchi, T. Kawakami, Y. Takano, Y. Kitagawa, Y. Yamashita, H. Fujita, *J. Quantum Chem.* **2002**, *90*, 370.

[7] S. Sinnecker, F. Neese, *J. Phys. Chem. A* **2006**, *110*, 12267.

[8] S. Stoll, A. Schweiger, *J. Magn. Reson.* **2006**, *178*, 42.
[9] T. Quintes, S. Weber, S. Richert, *J. Phys. Chem. A* **2025**, *129*, 3375.
[10] a) P. v. R. Schleyer, C. Maerker, A. Dransfeld, H. Jiao, N. J. R. van Eikema Hommes, *J. Am. Chem. Soc.* **1996**, *118*, 6317; b) Z. Chen, C. S. Wannere, C. Corminboeuf, R. Puchta, P. v. R. Schleyer, *Chem. Rev.* **2005**, *105*, 3842; c) K. Okamoto, S. Hatano, M. Abe, *J. Am. Chem. Soc.* **2024**; d) A. Rahalkar, A. Stanger, *"Aroma" package*; e) Z. Wang, *Chemistry* **2024**, *6*, 1692; f) A. Stanger, *Eur. J. Org. Chem.* **2020**, *2020*, 3120; g) A. Stanger, *Chemphyschem* **2023**, *24*, e202300080.
[11] a) University of Karlsruhe and Forschungszentrum Karlsruhe GmbH, 1989–2007, TURBOMOLE GmbH since 2007, *Turbomole V7.6 2021*; b) Jusélius Jonas, D. Sundholm, *Phys. Chem. Chem. Phys.* **1999**, *1*, 3429; c) J. Jusélius, D. Sundholm, J. Gauss, *J. Chem. Phys.* **2004**, *121*, 3952; d) J. Jusélius, R. Bast, H. Fliegl, D. Sundholm, M. Dimitrova, L. Wirz, V. Liegeois, C. Kumar, T. Kjærgaard, J. Kussmann, J. Pyykkö, T. Järvinen, J. Gauss, *The gauge including magnetically induced current density*.
[12] D. W. Szczepanik, M. Andrzejak, K. Dyduch, E. Żak, M. Makowski, G. Mazur, J. Mrozek, *Phys. Chem. Chem. Phys.* **2014**, *16*, 20514.
[13] a) R. Herges, D. Geuenich, *J. Phys. Chem. A* **2001**, *105*, 3214; b) D. Geuenich, K. Hess, F. Köhler, R. Herges, *Chem. Rev.* **2005**, *105*, 3758.
